# Acute Bilateral Vision Loss in Pregnancy: A Case of Posterior Reversible Encephalopathy Syndrome

**DOI:** 10.7759/cureus.111982

**Published:** 2026-07-03

**Authors:** Jai Chadha, Tanush Pandit, Anitha AM

**Affiliations:** 1 Obstetrics and Gynecology, Employees' State Insurance Corporation (ESIC) Model Hospital and Post Graduate Institute of Medical Science and Research (PGIMSR), Bangalore, IND

**Keywords:** magnetic resonance imaging, posterior leukoencephalopathy syndrome, posterior reversible encephalopathy syndrome, t2-weighted imaging, vasogenic edema

## Abstract

Posterior reversible encephalopathy syndrome (PRES) is a clinicoradiological entity characterized by headache, seizures, altered consciousness, and visual disturbances, with preeclampsia and eclampsia among its most common triggers. Although visual symptoms occur in roughly one-third of cases, acute cortical blindness as the predominant presenting feature in the absence of significant hypertension is uncommon and diagnostically challenging. We report a 27-year-old woman (gravida 2, abortion 1) at 36 weeks of gestation who presented with a one-day history of headache, a generalized tonic-clonic seizure, and sudden bilateral vision loss, with a blood pressure of 110/60 mmHg. Ophthalmological examination revealed no perception of light bilaterally with preserved pupillary reflexes and a normal fundus, localizing the deficit to the visual cortex. Investigations demonstrated mild anemia, thrombocytopenia, prolonged activated partial thromboplastin time (aPTT), and proteinuria. Magnetic resonance imaging revealed bilateral T2/fluid-attenuated inversion recovery (FLAIR) hyperintensities in the occipital lobes involving the primary visual cortex, confirming PRES. An emergency lower segment cesarean section was performed under general anesthesia for fetal distress. Postoperative autoimmune workup revealed strongly positive anti-nRNP and anti-Ro antibodies with a positive lupus anticoagulant. Management included the Zuspan magnesium sulfate regimen, hydroxychloroquine, aspirin, and bridging anticoagulation with enoxaparin to warfarin. Complete visual recovery was achieved by postoperative day 3. This case illustrates that acute bilateral cortical blindness may be a manifestation of PRES in eclampsia even without marked hypertension, consistent with the endothelial dysfunction model of pathogenesis. Timely multidisciplinary recognition and intervention facilitated complete recovery.

## Introduction

Posterior reversible encephalopathy syndrome (PRES), first described in 1996, is a reversible clinicoradiological condition characterized by acute neurological dysfunction and predominant posterior cerebral edema on imaging [1]. It is an important yet often underrecognized cause of sudden neurological and visual deficits.

The exact pathophysiology has not yet been fully established. Three main mechanisms have been proposed: cerebral vasoconstriction causing transient ischemia, failure of cerebral autoregulation leading to vasogenic edema, and endothelial dysfunction with disruption of the blood-brain barrier, resulting in leakage of fluid and proteins into the brain parenchyma [2]. An alternative hypothesis proposes that widespread endothelial dysfunction due to underlying connective tissue disorder, triggered by systemic inflammation, hypoxia, or other circulating factors, results in cerebral vasoconstriction and reduced perfusion, ultimately leading to cerebral edema [3]. This mechanism may be particularly relevant in our patient, whose normotensive presentation suggests that endothelial injury, rather than severe hypertension, was the predominant driver of PRES. 

PRES is most commonly observed in women with hypertensive disorders of pregnancy, especially in patients with eclampsia (98%). It has also been reported in patients undergoing bone marrow transplantation (2.7-25%), as well as in those with end-stage renal disease and autoimmune conditions (0.4-0.8%) and recipients of solid organ transplantation (0.4-0.6%). In obstetric patients, endothelial injury and hemodynamic instability are primary factors, and it has been observed that PRES may occur even without severe hypertension (20-30%) [4]. 

Visual symptoms are observed in about 39% of patients with PRES ranging from blurred vision and field defects to cortical blindness, which could occur despite normal anterior segment and fundus because the lesion is retrochiasmal [5]. As per the literature, vision usually improves within hours to days after treatment, with recovery often occurring within four hours to eight days [6].

This case describes an unusual presentation of PRES in a patient with a suspected underlying connective tissue disorder, isolated prolongation of activated partial thromboplastin time (aPTT), normotension at presentation, and eclampsia, who subsequently developed acute bilateral visual loss lasting 24-36 hours. It highlights the diverse clinical spectrum of PRES and emphasizes that the diagnosis should be considered even in the absence of severe hypertension. This report also provides an overview of the established and emerging risk factors associated with PRES, underscoring the importance of early recognition, prompt neuroimaging, and timely multidisciplinary management to achieve favorable neurological and visual outcomes.

## Case presentation

A 27-year-old woman (gravida 2, abortion 1) at 36 weeks of gestation presented to the obstetric triage with sudden painless bilateral vision loss following a generalized tonic-clonic seizure at home one hour earlier. She reported a headache the previous day, relieved with symptomatic treatment at a local hospital. Her antenatal history was unremarkable, with no history of hypertension, diabetes mellitus, renal, cardiac, or thyroid disease, chronic medication use, or previous surgery.

On examination, she was conscious, oriented, and without postictal confusion. Mild pallor was present, but there was no pedal edema. Pulse rate was normal, and blood pressure was 110/60 mmHg. Cardiovascular, respiratory, and neurological examinations were normal except for complete visual loss. Laboratory investigations revealed mild anemia (Hb 8.7 g/dL), prolonged aPTT (70 s), thrombocytopenia (75,000/µL), and elevated urine protein-creatinine ratio (2.1). Liver and renal function tests were within normal limits.

Obstetric examination revealed a uterus corresponding to approximately 30 weeks of gestation (suggestive of fetal growth restriction) with cephalic presentation, a relaxed uterus, a fetal heart rate of 140-150 bpm, and an uneffaced cervix with a Bishop score of 6. Ultrasonography demonstrated a live singleton fetus in cephalic presentation weighing 1.6 kg (<10th percentile), a cerebroplacental ratio of less than 1, and an amniotic fluid index of 4 cm. Ophthalmic examination showed negative light perception bilaterally, with normal anterior segments and briskly reactive pupils. Fundus examination showed normal discs, preserved arteriovenous ratio, and intact macular foveal reflex.

Magnetic resonance imaging (MRI) of the brain (Figure 1) done upon admission demonstrated multiple cortical and gyral T2/fluid-attenuated inversion recovery (FLAIR) hyperintensities involving both cerebral hemispheres, predominantly the occipital lobes (right greater than left), with additional involvement of the frontal gyri, pre- and post-central gyri, and bilateral basal ganglia, consistent with vasogenic edema and PRES.

**Figure 1:**
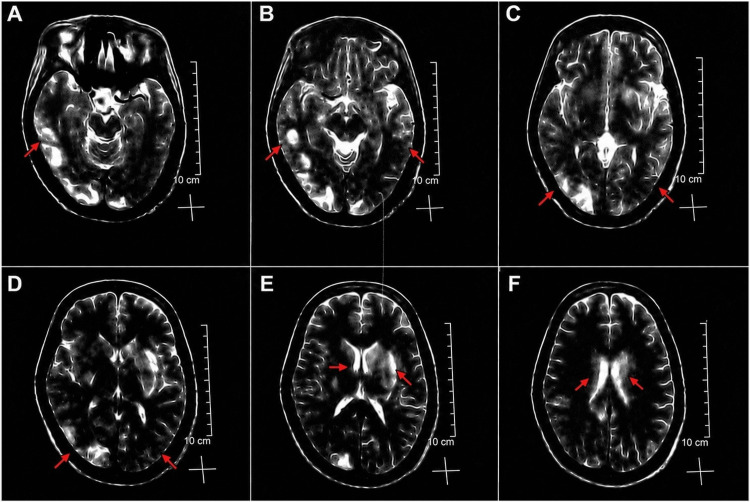
Axial T2-weighted magnetic resonance images of the brain (A-F) showing bilateral parietooccipital hyperintensities with cerebellar involvement, consistent with posterior reversible encephalopathy syndrome Red arrows indicate bilateral parietooccipital cortical and subcortical T2/fluid-attenuated inversion recovery (FLAIR) hyperintensities consistent with vasogenic edema.

A diagnosis of PRES presenting as acute cortical blindness in the setting of atypical (normotensive) antepartum eclampsia was made. Differential diagnoses, including cerebral venous sinus thrombosis and occipital lobe infarction, were considered and excluded based on the clinical presentation, ophthalmic examination, and characteristic MRI findings.

The patient was stabilized with intravenous magnesium sulfate according to the Zuspan regimen, consisting of a 4-gram intravenous loading dose followed by a continuous infusion of 1 gram per hour, and administered betamethasone for fetal lung maturation. Labor induction with dinoprostone gel was planned after Bishop score assessment. In view of deranged hematological parameters, two units of fresh frozen plasma were transfused preoperatively, with one additional unit intraoperatively, along with two units of platelets and one unit of packed red blood cells as required postoperatively.

Subsequently, an emergency lower segment cesarean section under general anesthesia was performed in view of fetal heart rate abnormalities (late decelerations). Scanty meconium-stained liquor and a posterior calcified placenta were noted, and a live male infant weighing 1.65 kg was delivered by vertex extraction and transferred to the neonatal intensive care unit.

Postoperative ophthalmic evaluation showed normal anterior and posterior segment findings without papilledema. Vision improved to counting fingers at three meters and returned to near normal by postoperative day 3.

The patient received magnesium sulfate as per the Zuspan regimen for 24 hours after the delivery, antibiotics, and supportive care. Further evaluation of the isolated prolonged aPTT revealed strongly positive anti-nRNP and anti-Ro antibodies. Lupus anticoagulant was positive, whereas anticardiolipin and anti-β2 glycoprotein I antibodies were negative. Rheumatology consultation was obtained on postoperative day 2, and hydroxychloroquine (200 mg) was initiated. Anticoagulation with low-molecular-weight heparin and warfarin bridging was commenced, with plans to continue warfarin and low-dose aspirin. The patient improved steadily and was discharged seizure-free, hemodynamically stable, and with improving vision, with follow-up planned in obstetrics, neurology, ophthalmology, rheumatology, and gastroenterology.

## Discussion

The present case is notable for sudden bilateral painless loss of vision with preserved pupillary reflexes and a normal anterior segment and fundus, suggesting cortical blindness secondary to PRES rather than ocular pathology. This uncommon presentation may delay diagnosis because ocular examination remains normal despite profound visual loss. Similar rare presentations were described by Waghamare et al. in severe preeclampsia, where cortical blindness may be the presenting feature in up to 15% of cases of PRES-associated visual involvement [7]. The timing and spectrum in our patient are consistent with PRES as reported in the literature. In the eclampsia series by Karuppannasamy et al., seizures occurred in approximately 80% of patients with visual disturbances including cortical blindness [8].

An important feature of this case is the occurrence of PRES despite the absence of hypertension at presentation, as PRES is often suspected only with severe hypertension. Clinical reviews indicate that approximately 20-30% of PRES patients may be normotensive, emphasizing endothelial dysfunction and impaired autoregulation as key mechanisms [4]. 

A particularly noteworthy finding was the autoimmune profile, with strongly positive anti-nRNP and anti-Ro antibodies and a positive lupus anticoagulant. Although these findings alone do not confirm systemic lupus erythematosus or antiphospholipid syndrome, they may have contributed to the development of PRES. Valdez-López et al. identified lupus as the autoimmune disease most commonly associated with PRES, with autoimmune abnormalities reported in up to 50% of patients [9]. Similarly, Machiraju et al. reported PRES as the first manifestation of mixed connective tissue disease, supporting a possible autoimmune contribution in our patient [10]. The presence of lupus anticoagulant with anti-Ro and anti-nRNP antibodies raised suspicion for an underlying autoimmune prothrombotic state. Although the patient did not meet the diagnostic criteria for antiphospholipid syndrome, these findings prompted treatment with hydroxychloroquine, low-dose aspirin, and enoxaparin bridged to warfarin, along with planned rheumatology follow-up.

Comparable reports, including Gould et al., described acute bilateral visual loss with normal ocular findings, supporting the retrochiasmal origin of vision loss; visual acuity may range from perception of light to complete blindness, with rapid reversibility after treatment [11].

Radiologically, PRES typically demonstrates vasogenic edema in the parietooccipital regions in nearly 70-80% of cases, although atypical involvement of the frontal lobes, cerebellum, or brainstem may occur in up to 30% of patients [12]. Management aligns with current literature emphasizing seizure prophylaxis and treatment of the underlying cause. As noted by Nasereddin et al., with timely intervention, improvement often begins within 24-48 hours, and visual recovery usually occurs within days [13].

## Conclusions

This case underscores the importance of considering PRES in pregnant or postpartum patients presenting with acute bilateral vision loss, particularly when cortical blindness occurs in the presence of preserved pupillary reflexes and a normal ophthalmic examination. Although PRES is classically associated with severe hypertension and eclampsia, atypical presentations may occur in normotensive patients or those with underlying conditions associated with endothelial dysfunction, increasing the risk of delayed diagnosis. Prompt neuroimaging is therefore essential to distinguish PRES from primary ocular and other neurological causes of visual loss. 

Early recognition and timely multidisciplinary management, including seizure control, optimization of blood pressure, treatment of the underlying precipitating factor, and appropriate obstetric intervention, are crucial for preventing irreversible neurological injury and ensuring favorable visual outcomes. This case further highlights the potential role of connective tissue disorders and associated endothelial dysfunction in the pathogenesis of PRES, broadening the spectrum of recognized risk factors. Greater awareness of these uncommon presentations may facilitate earlier diagnosis, improve patient outcomes, and contribute to a better understanding of the diverse clinical manifestations of PRES. 
